# The value of combined SATB2 and CDX2 detection in the differential diagnosis of colorectal adenocarcinoma

**DOI:** 10.1097/MD.0000000000050180

**Published:** 2026-08-28

**Authors:** Liping Wang, Hongjun Li, Liping Feng

**Affiliations:** a Shanghai Jince Medical Laboratory Co., Ltd, Shanghai City, China; b Department of Colorectal and Anal Surgery, Chifeng Municipal Hospital, Chifeng Clinical Medical School of Inner Mongolia Medical University, Chifeng City, Inner Mongolia Autonomous Region, China; c Department of Oncology, Affiliated Hospital of Guangdong Medical University, Zhanjiang City, Guangdong Province, China.

**Keywords:** adenocarcinoma, CDX2, colorectal neoplasms, diagnostic efficacy, differential diagnosis, immunohistochemistry, SATB2

## Abstract

This study evaluates the diagnostic value of SATB2 and CDX2, used individually and in combination, in the immunohistochemical differential diagnosis of colorectal adenocarcinoma. A retrospective case-control diagnostic accuracy study was conducted, including 122 patients with histologically confirmed colorectal adenocarcinoma or non-colorectal adenocarcinoma. Immunohistochemical staining for SATB2 and CDX2 was performed on all cases, followed by semiquantitative interpretation. Using histopathological diagnosis as the gold standard, the diagnostic efficacy of each test index (SATB2 positive, CDX2 positive, either positive, and both positive) was calculated. SATB2 and CDX2 were highly expressed in colorectal adenocarcinoma (positive rates of 88.2% and 86.3%, respectively), which was significantly higher than in the control group (7.0% and 19.7%, *P* < .001). SATB2 used alone demonstrated the optimal diagnostic discrimination among single markers (AUC = 0.916, specificity 93.0%). Among the combined strategies, the “either positive” rule achieved the highest sensitivity (94.1%), whereas the “both positive” rule showed the highest specificity (98.6%) and overall discriminative ability (AUC = 0.938). PPV and NPV values were interpreted only within this case-control study population and should not be considered direct estimates of performance in populations with different disease prevalence. CDX2 showed the lowest specificity (60.0%) in differentiating gastric adenocarcinoma, whereas the “both positive” strategy maintained high diagnostic reliability across all differential scenarios. The sensitivity of both markers was significantly reduced in poorly differentiated colorectal adenocarcinoma. SATB2 is an excellent single diagnostic marker. The combined application of SATB2 and CDX2 offers complementary advantages: “either positive” is suitable for exclusion diagnosis, while “both positive” is suitable for confirmation diagnosis. This combination is an effective tool for the differential diagnosis of colorectal adenocarcinoma, although caution is needed when diagnosing poorly differentiated carcinomas.

## 1. Introduction

Colorectal Cancer (CRC) is one of the malignant tumors with high morbidity and mortality rates worldwide. With changes in the global economy and lifestyle, the incidence of colorectal cancer is showing an increasing trend year by year.^[1,2]^ Accurate diagnosis and differential diagnosis are crucial for the treatment and prognostic evaluation of colorectal cancer. Due to the morphological overlap between colorectal cancer and adenocarcinomas from other origins, the use of immunohistochemical (IHC) markers provides significant assistance in clinical pathological diagnosis.^[3,4]^

SATB2 (special AT-rich sequence-binding protein 2) and CDX2 (caudal type homeobox 2) are immunohistochemical markers that have gained increasing attention in the diagnosis of colorectal cancer in recent years.^[5]^ SATB2 is a nuclear matrix-associated protein involved in regulating gene expression, playing an important role particularly in colon development.^[6]^ CDX2 is a crucial homeobox gene whose key function is to maintain the normal function of the intestinal epithelium during the differentiation of intestinal epithelial cells.^[7]^ The expression of SATB2 and CDX2 in colorectal cancer has been demonstrated by multiple studies to possess high sensitivity and specificity.

Although SATB2 and CDX2 each perform well in the diagnosis of colorectal cancer when used alone, there are still certain limitations to their individual diagnostic efficacy. Recent research indicates that when SATB2 is combined with CDX2, their complementary advantages can be better utilized, enhancing the sensitivity and specificity of diagnosis.^[8]^ Particularly in diagnostically challenging cases, the combined application of these 2 markers may improve the accuracy of differential diagnosis. For instance, when differentiating colorectal cancer from other types of adenocarcinoma, such as gastric adenocarcinoma or ovarian mucinous adenocarcinoma, the combined use of SATB2 and CDX2 can provide clearer diagnostic results.^[9,10]^

Despite this, existing literature mostly focuses on evaluating the diagnostic efficacy of SATB2 or CDX2 as individual markers, while research on the comprehensive efficacy of their combined application, especially its performance in different subgroups (such as poorly differentiated colorectal adenocarcinoma), remains insufficient.^[11]^ This study aims to systematically evaluate the clinical value of SATB2 and CDX2, used individually and in combination, in the differential diagnosis between colorectal adenocarcinoma and other types of adenocarcinoma through a retrospective case-control study.

The results of this study will not only help clarify the diagnostic value of SATB2 and CDX2 in clinical practice but also provide clinical pathologists with more reference information regarding the application of these immunohistochemical markers in differentiating colorectal cancer from other adenocarcinomas, further promoting their application in clinical diagnosis.

## 2. Materials and methods

### 2.1. Study design

This study was approved by the Ethics Committee of Affiliated Hospital of Guangdong Medical University. This study is a retrospective, diagnostic study designed to systematically evaluate and compare the value of the 2 transcription factors, SATB2 and CDX2, used individually and in combination in immunohistochemical staining for the differential diagnosis of colorectal adenocarcinoma. Cases pathologically diagnosed between December 2023 and December 2025 were retrospectively reviewed and collected according to predefined eligibility criteria. By comparing with the established “gold standard” (including tissue morphology, comprehensive clinical history, and other immunohistochemical markers), the sensitivity, specificity, positive predictive value, negative predictive value, and diagnostic accuracy of SATB2 and CDX2, detected individually and in combination, will be calculated to clarify their application efficacy.

### 2.2. Study groups

A retrospective case-control design based on the final pathological diagnosis (gold standard) was used for grouping.

The case group (colorectal adenocarcinoma group) will include primary colorectal adenocarcinoma cases confirmed by histopathology, used to evaluate the detection sensitivity of SATB2 and CDX2.

The control group (non-colorectal adenocarcinoma group) will include other types of adenocarcinoma that require differential diagnosis from colorectal cancer. This group will be further subdivided into several subgroups to evaluate the specificity of the markers, mainly including the upper gastrointestinal/pancreaticobiliary adenocarcinoma subgroup (comprising gastric, esophageal, pancreatic, and biliary tract adenocarcinomas), the gynecological/pelvic adenocarcinoma subgroup (primarily ovarian mucinous adenocarcinoma), the lung adenocarcinoma subgroup, and other adenocarcinoma subgroups with potential cross-expression (such as bladder adenocarcinoma, etc).

### 2.3. Inclusion and exclusion criteria

#### 2.3.1. Inclusion criteria

Cases meeting all of the following conditions will be included in this study: cases with archived paraffin-embedded tissue blocks obtained via surgical resection or biopsy; cases reviewed independently by 2 pathologists with associate chief physician titles or higher, and definitively diagnosed as primary colorectal adenocarcinoma (case group) or adenocarcinoma of the corresponding organ origin (control group) according to the World Health Organization Classification of Digestive System Tumours, 5th edition; cases with complete clinical data (including age, sex, primary tumor site, and pathological type) and pathological information; and archived tissue blocks containing an adequate amount of tumor cells (generally recommended that the tumor cell area accounts for ≥10%), with well-fixed and well-preserved tissue suitable for immunohistochemical staining.

#### 2.3.2. Exclusion criteria

Cases meeting any of the following conditions will be excluded: poorly fixed tissue samples, severe autolysis, or insufficient tumor cell content that cannot meet the requirements for staining or interpretation; missing key clinical or pathological information, preventing the determination of the final diagnosis or primary site; Patients in the case group who received neoadjuvant radiotherapy or chemotherapy for colorectal cancer before surgery (patients in the control group who received antitumor therapy for the target tumor before biopsy or surgery); metastatic tumors where the tissue cannot be obtained or cannot be clearly associated with the primary lesion for analysis; and synchronous multiple primary cancers, making it difficult to define the main study lesion.

### 2.4. Sample size estimation

The sample size for this study was calculated based on the standard formula for diagnostic tests. Referring primarily to reliable published literature, the baseline expected values for the target diagnostic indices were set: the expected diagnostic sensitivity (Sen) for SATB2 and CDX2 in colorectal cancer (case group) was assumed to be 0.90, and the expected diagnostic specificity (Spe) in non-colorectal cancer (control group) was assumed to be 0.85. The significance level was set at α = 0.05 (two-sided test), and the allowable error δ = 0.10.

Based on this, the required minimum sample sizes for the case group and control group were estimated separately:

**Case group (colorectal adenocarcinoma):** The sample size was calculated using the formula: n_case = Zα/2^2^ × Sen(1 − Sen)/δ^2^. Substituting Zα/2 = 1.96, the calculation yielded a minimum of 35 cases.

**Control group (non-colorectal carcinomas for differential diagnosis):** The sample size was calculated using the formula: n_control = Zα/2^2^ × Spe(1 − Spe)/δ^2^. The calculation yielded a minimum of 49 cases for the control group.

To fully account for potential invalid specimens in a retrospective study (e.g., insufficient tissue, poor staining quality, missing key data) and to meet the basic requirements for meaningful subgroup analysis of the control group (such as distinguishing different primary sites), the theoretically calculated sample sizes were expanded by approximately 20%. Therefore, the total planned sample size for this study is 122 cases, comprising 51 cases in the colorectal adenocarcinoma group and 71 cases in the non-colorectal cancer control group requiring differentiation. The control group will be further stratified according to common differential diagnosis types, including gastric adenocarcinoma, ovarian mucinous adenocarcinoma, and lung adenocarcinoma, with no fewer than 15 cases per subgroup to ensure basic statistical power for each subgroup analysis. Specific baseline information is shown in Table 1.

**Table 1:** Baseline clinicopathological characteristics of the study subjects (n = 122).

Group	Cases n (%)	Primary tumor site (n, %)	Pathological type (n, %)	Differentiation grade (n, %)
Colorectal adenocarcinoma group	51 (41.8%)	Colon: 32 (62.7%) Rectum: 19 (37.3%)	Adenocarcinoma: 46 (90.2%) Mucinous adenocarcinoma: 5 (9.8%)	Well-differentiated: 12 (23.5%) Moderately-differentiated: 30 (58.8%) Poorly-differentiated: 9 (17.7%)
Non-colorectal adenocarcinoma control group	71 (58.2%)			
Gastric adenocarcinoma subgroup	20 (16.4%)	Stomach: 20 (100.0%)	Adenocarcinoma: 18 (90.0%) Signet ring cell carcinoma: 2 (10.0%)	Well-differentiated: 3 (15.0%) Moderately-differentiated: 12 (60.0%) Poorly-differentiated: 5 (25.0%)
Ovarian mucinous adenocarcinoma subgroup	18 (14.8%)	Ovary: 18 (100.0%)	Mucinous adenocarcinoma: 18 (100.0%)	Moderately-differentiated: 15 (83.3%) Poorly-differentiated: 3 (16.7%)
Lung adenocarcinoma subgroup	18 (14.8%)	Lung: 18 (100.0%)	Adenocarcinoma: 18 (100.0%)	Moderately-differentiated: 11 (61.1%) Poorly-differentiated: 7 (38.9%)
Other adenocarcinoma subgroup	15 (12.3%)	Pancreas: 6 (40.0%) Endometrium: 5 (33.3%) Other: 4 (26.7%)	Adenocarcinoma: 15 (100.0%)	Moderately-differentiated: 10 (66.7%) Poorly-differentiated: 5 (33.3%)
Total	122 (100.0%)	**–**		

“Other” refers to bladder adenocarcinoma (2 cases) and cholangiocarcinoma (2 cases).

### 2.5. SATB2 and CDX2 detection methods

#### 2.5.1. Immunohistochemical staining

All paraffin-embedded tissue sections included in the study will undergo immunohistochemical staining for SATB2 and CDX2. Archived paraffin blocks of all enrolled cases will be retrieved from the pathology department’s slide library. Staining will be performed using the EnVision 2-step method on an automated staining platform to ensure standardized operation. The specific steps are as follows: Blocks containing representative tumor areas will be selected and serially sectioned (thickness 4 μm). After deparaffinization and rehydration, heat-induced epitope retrieval will be performed using the retrieval solution (ethylenediaminetetraacetic acid buffer, pH 9.0) and conditions recommended in the manuals for the SATB2 antibody (clone: EP281) and the CDX2 antibody (clone: EPR2764Y), respectively. Following retrieval, the corresponding primary antibody working solution will be applied, and sections will be incubated overnight at 4°C. The next day, sections will be incubated with horseradish peroxidase-labeled secondary antibody polymer at room temperature, visualized with DAB chromogen, counterstained with hematoxylin, dehydrated through a graded ethanol series, cleared in xylene, and mounted with neutral balsam. Positive controls (known positive colorectal cancer tissue) and negative controls (replacing the primary antibody with phosphate buffered saline) will be included in each staining batch to verify the validity and specificity of the staining process.

#### 2.5.2. Result interpretation and quantification

The immunohistochemical results for SATB2 and CDX2 will be interpreted independently by 2 senior attending pathologists from the pathology department under double-blind conditions (unaware of the clinicopathological grouping information of the cases). The positive staining signal is localized in the nucleus, appearing as yellow to brownish-yellow granular coloration. Interpretation will employ a semiquantitative scoring method, evaluating both the percentage of positive cells and the intensity of nuclear staining.^[12]^

1. **Percentage of positive cells score:** under low-power magnification (×40), 5 representative fields with dense tumor cells and a clear staining background will be selected. Switching to high-power magnification (×200), the percentage of positive tumor cells in each field will be counted. The average percentage from the 5 fields will be taken as the final percentage for the case and scored according to the following criteria: <5% positive cells = 0 points; 5% to 25% = 1 point; 26% to 50% = 2 points; 51% to 75% = 3 points; >75% = 4 points.
2. **Staining intensity score:** under high-power magnification (×200), the average color intensity of positive nuclei will be assessed and scored as follows: no coloration = 0 points; light yellow = 1 point; brownish-yellow = 2 points; dark brown = 3 points.
3. **Comprehensive judgment:** the percentage of positive cells score will be multiplied by the staining intensity score to obtain a final total score. Based on commonly used clinical thresholds and literature references, a total score of ≥2 points will be defined as positive expression in this study, recorded as SATB2(+) or CDX2(+); a total score of <2 points will be defined as negative expression, recorded as SATB2(−) or CDX2(−). In case of discordance between the interpretations of the 2 pathologists, the final result will be determined through discussion during joint slide review or arbitration by a third pathologist with an associate chief physician title. For combined detection, the expression status of SATB2 and CDX2 will be recorded separately, and the diagnostic value of their combined diagnostic patterns (such as both positive, either positive, etc) will be analyzed.

### 2.6. Data collection

In addition to the immunohistochemical results for SATB2 and CDX2, data for all included cases will be systematically collected through retrospective review of the hospital’s pathology information system and electronic medical records. Collected data will include demographic information (age, sex), clinical data (primary tumor site, clinical diagnosis), and pathological data (specimen type, final histopathological diagnosis and subtype, tumor differentiation grade, and pTNM (tumor node metastasis) stage for the colorectal adenocarcinoma group). Based on the final histopathological diagnosis (gold standard) and immunohistochemical results, diagnostic efficacy parameters will be calculated. All data will be double-checked by 2 individuals before entry into a dedicated database to ensure accuracy and completeness.

### 2.7. Statistical analysis

SPSS 26.0 software (SPSS Inc.) will be used for statistical analysis. Measurement data conforming to a normal distribution will be expressed as mean ± standard deviation (Mean ± SD), and comparisons between groups will be performed using the independent samples *t*-test. Data not conforming to a normal distribution will be expressed as median (interquartile range) [M (P25, P75)], and comparisons between groups will be performed using the Mann–Whitney *U* test. Categorical data will be expressed as number of cases (percentage; n, %), and comparisons between groups will be performed using the Chi-square test or Fisher’s exact test. Receiver operating characteristic curve analysis will be used to evaluate the comprehensive diagnostic efficacy of each test index, calculating the area under the curve (AUC) and its 95% confidence interval (CI), and determining the optimal cutoff value. Binary logistic regression analysis will be used to evaluate the independent predictive value of each marker for colorectal adenocarcinoma, calculating the odds ratio and its 95% CI. All statistical tests will be 2-sided, and a *P*-value < .05 will be considered statistically significant. For clinicopathological subgroup analyses, sensitivity was calculated as the proportion of marker-positive tumors among colorectal adenocarcinoma cases in each subgroup and was reported with exact 95% confidence intervals (CI) using the Clopper–Pearson method. Differences in sensitivity between subgroups were assessed using the chi-square test or Fisher’s exact test, as appropriate. Because these analyses were exploratory and some subgroups contained small numbers of cases, the results were interpreted cautiously.

## 3. Results

### 3.1. Baseline characteristics of the study population

A total of 122 patients were included in this study, comprising 51 cases in the colorectal adenocarcinoma group and 71 cases in the non-colorectal adenocarcinoma control group (Table 2). There were no statistically significant differences between the 2 groups in terms of age, sex, or specimen type (*P* > .05). The proportion of mucinous adenocarcinoma in the control group (19.7%) was higher than that in the colorectal adenocarcinoma group (9.8%). The most significant difference between the 2 groups was in the degree of differentiation: the proportion of poorly differentiated tumors in the control group was significantly higher than that in the colorectal adenocarcinoma group (50.7% vs 17.6%, *P* < .001). The colorectal adenocarcinoma group predominantly consisted of patients with stage II–III disease (74.5%).

**Table 2:** Comparison of baseline characteristics between the colorectal adenocarcinoma group and the non-colorectal adenocarcinoma control group (n = 122).

Characteristic	Colorectal adenocarcinoma group (n = 51)	Non-colorectal adenocarcinoma control Group (n = 71)	Statistic	*P*-value
Age [yr, M (P25, P75)]	65.0 (58.0, 72.0)	61.0 (52.0, 68.0)	Z = -1.96	.05
Sex (n, %)			χ^2^ = 1.87	.171
Male	29 (56.9%)	32 (45.1%)		
Female	22 (43.1%)	39 (54.9%)		
Specimen type (n, %)			χ^2^ = 0.60	.438
Surgical resection	48 (94.1%)	64 (90.1%)		
Biopsy	3 (5.9%)	7 (9.9%)		
Pathological type (n, %)				
Adenocarcinoma (classical)	46 (90.2%)	57 (80.3%)		
Mucinous adenocarcinoma	5 (9.8%)	14 (19.7%)		
Differentiation Grade (n, %)			χ^2^ = 15.32	<.001
Well/moderately-differentiated	42 (82.4%)	35 (49.3%)		
Poorly-differentiated	9 (17.6%)	36 (50.7%)		
Colorectal cancer TNM stage (n, %)				
Stage I	8 (15.7%)	–		
Stage II	18 (35.3%)	–		
Stage III	20 (39.2%)	–		
Stage IV	5 (9.8%)	–		

TNM = tumor node metastasis.

### 3.2. SATB2 and CDX2 show significantly different expression patterns between the colorectal adenocarcinoma group and the control group

Both markers were highly expressed in colorectal adenocarcinoma, with positivity rates of 88.2% and 86.3%, respectively, and a double positivity rate of 80.4% (Table 3). Their mean comprehensive scores were 6.8 ± 3.1 and 5.2 ± 3.0, respectively (Table 4). In the non-colorectal adenocarcinoma control group, the positivity rates and expression intensities of both markers were significantly lower than those in the colorectal adenocarcinoma group (*P* < .001). Specifically, the positivity rate of SATB2 was extremely low in the control group and its subgroups (5.0–11.1%), with mean comprehensive scores ranging only from 0.5 to 0.9. False positives for CDX2 were mainly concentrated in the gastric adenocarcinoma subgroup, where the positivity rate reached 40.0%, and the mean comprehensive score (3.5 ± 3.0) was also significantly higher than in other control subgroups.

**Table 3:** Expression of SATB2 and CDX2 in each tumor group and comparison with the colorectal adenocarcinoma group.

Group (n)	SATB2 positive (n, %)	χ^2^/Fisher value	*P*-value	CDX2 positive (n, %)	χ^2^/Fisher value	*P*-value	Double positive (SATB2+/CDX2+; n, %)	χ^2^/Fisher value	*P*-value
Colorectal adenocarcinoma group (51)	45 (88.2)			44 (86.3)			41 (80.4)		
Non-colorectal adenocarcinoma control group (71)	5 (7.0)	73.25	<.001	14 (19.7)	58.92	<.001	1 (1.4)	89.16	<.001
Gastric adenocarcinoma subgroup (20)	1 (5.0)	41.76	<.001	8 (40.0)	15.98	<.001	0 (0.0)	43.28	<.001
Ovarian mucinous adenocarcinoma subgroup (18)	1 (5.6)	40.12	<.001	3 (16.7)	31.52	<.001	0 (0.0)	41.54	<.001
Lung adenocarcinoma subgroup (18)	2 (11.1)	35.67	<.001	2 (11.1)	35.67	<.001	1 (5.6)	31.14	<.001
Other adenocarcinoma subgroup (15)	1 (6.7)	38.45	<.001	1 (6.7)	38.45	<.001	0 (0.0)	40	<.001

CDX2 = caudal type homeobox 2, SATB2 = special AT-rich sequence-binding protein 2.

**Table 4:** Comparison of immunohistochemical comprehensive Scores for SATB2 and CDX2 between groups (Mean ± SD).

Group (n)	Index	Comprehensive score (Mean ± SD)	Comparison with colorectal cancer group	
			*t*-value	*P*-value
Colorectal adenocarcinoma group (51)	SATB2	6.8 ± 3.1	–	–
	CDX2	5.2 ± 3.0	–	–
Non-colorectal adenocarcinoma control group (71)	SATB2	0.7 ± 1.5	15.32	<.001
	CDX2	1.8 ± 2.4	7.89	<.001
Gastric adenocarcinoma subgroup (20)	SATB2	0.5 ± 1.2	10.45	<.001
	CDX2	3.5 ± 3.0	2.18	.034
Ovarian mucinous adenocarcinoma subgroup (18)	SATB2	0.9 ± 1.4	9.67	<.001
	CDX2	2.1 ± 2.2	4.12	<.001
Lung adenocarcinoma subgroup (18)	SATB2	0.6 ± 1.1	10.12	<.001
	CDX2	1.0 ± 1.5	6.78	<.001
Other adenocarcinoma subgroup (15)	SATB2	0.6 ± 1.3	9.23	<.001
	CDX2	1.1 ± 1.6	6.05	<.001

CDX2 = caudal type homeobox 2, SATB2 = special AT-rich sequence-binding protein 2, SD = standard deviation.

### 3.3. Diagnostic efficacy of SATB2 and CDX2

Both SATB2 and CDX2, used individually and in combination, demonstrated good differential diagnostic efficacy (Table 5). Among the single indicators, SATB2 exhibited the highest overall diagnostic accuracy, with an AUC of 0.916 (95% CI, 0.861–0.971), and the highest specificity (93.0%) and positive predictive value (90.0%). The combined strategies optimized diagnostic characteristics: the “either positive” strategy increased sensitivity to 94.1% and achieved a negative predictive value of 94.7%, making it suitable for exclusion diagnosis; whereas the “both positive” strategy achieved the highest overall discriminative ability (AUC = 0.938), along with near-perfect specificity (98.6%) and positive predictive value (97.6%), offering the highest value for confirmation diagnosis.

**Table 5:** Diagnostic efficacy of SATB2 and CDX2, used Individually and in combination, in differentiating colorectal adenocarcinoma.

Test index	AUC (95% CI)	Standard error	*P*-value	Optimal cutoff value	Sensitivity (%)	Specificity (%)	Positive predictive value (%)	Negative predictive value (%)	Accuracy (%)
SATB2 positive	0.916 (0.861–0.971)	0.028	<.001	≥2 points	88.2	93.0	90.0	91.8	90.9
CDX2 positive	0.852 (0.781–0.923)	0.036	<.001	≥2 points	86.3	80.3	75.9	89.1	82.8
Either SATB2 or CDX2 positive	0.884 (0.823–0.945)	0.031	<.001	–	94.1	74.6	72.7	94.7	83.6
Both SATB2 and CDX2 positive	0.938 (0.892–0.984)	0.023	<.001	–	80.4	98.6	97.6	88.5	90.1

CDX2 = caudal type homeobox 2, CI = confidence interval, SATB2 = special AT-rich sequence-binding protein 2.

### 3.4. Diagnostic efficacy analysis by tumor subgroup

The efficacy of the various test indices differed in the differential diagnosis of different tumor types (Table 6). When differentiating gastric adenocarcinoma, CDX2 showed the lowest specificity (60.0%), leading to a significant decrease in the specificity (55.0%) and positive predictive value (67.9%) of the “either positive” strategy. In contrast, the “both positive” strategy achieved 100% for both specificity and positive predictive value. When differentiating ovarian mucinous adenocarcinoma and lung adenocarcinoma, all indices demonstrated relatively high specificity (>77.8%). The “both positive” strategy exhibited the optimal diagnostic reliability for confirmation across all differential diagnosis scenarios.

**Table 6:** Subgroup diagnostic efficacy of SATB2 and CDX2 in differentiating colorectal adenocarcinoma from specific tumor types.

Tumor type for differential diagnosis	Test index	Sensitivity (%)	Specificity (%)	Positive predictive value (%)	Negative predictive value (%)
Gastric adenocarcinoma (n = 20)	SATB2 positive	88.2	95.0	94.7	88.9
	CDX2 positive	86.3	60.0	68.3	81.8
	Either positive	94.1	55.0	67.9	91.7
	Both positive	80.4	100.0	100.0	76.9
Ovarian mucinous adenocarcinoma (n = 18)	SATB2 positive	88.2	94.4	95.5	85.0
	CDX2 positive	86.3	83.3	87.5	81.8
	Either positive	94.1	77.8	84.6	91.3
	Both positive	80.4	100.0	100.0	76.9
Lung adenocarcinoma (n = 18)	SATB2 positive	88.2	88.9	90.0	86.7
	CDX2 positive	86.3	88.9	89.7	85.2
	Either positive	94.1	83.3	86.4	92.3
	Both positive	80.4	94.4	95.2	77.1

CDX2 = caudal type homeobox 2, SATB2 = special AT-rich sequence-binding protein 2.

### 3.5. Diagnostic efficacy of clinical pathological subgroups

The sensitivity of SATB2, CDX2, and their combined “both positive” rule was further evaluated across clinicopathological subgroups of the 51 colorectal adenocarcinoma cases (Table 7). Sensitivity estimates are reported together with the number of marker-positive cases, subgroup denominators, and exact 95% CI.

**Table 7:** Sensitivity of SATB2 and CDX2 according to clinicopathological characteristics of colorectal adenocarcinoma.

Clinicopathological characteristic	SATB2 positive, n/N (%)	95% CI	*P*-value	CDX2 positive, n/N (%)	95% CI	*P*-value	Both positive, n/N (%)	95% CI	*P*-value
Age (yr)			.936			.746			.946
≤65	23/26 (88.5)	69.8–97.6		22/26 (84.6)	65.1–95.6		21/26 (80.8)	60.6–93.4	
>65	22/25 (88.0)	68.8–97.5		22/25 (88.0)	68.8–97.5		20/25 (80.0)	59.3–93.2	
Sex			.695			.508			.364
Male	25/29 (86.2)	68.3–96.1		24/29 (82.8)	64.2–94.2		22/29 (75.9)	56.5–89.7	
Female	20/22 (90.9)	70.8–98.9		20/22 (90.9)	70.8–98.9		19/22 (86.4)	65.1–97.1	
Tumor site			.669			.746			.367
Colon	29/32 (90.6)	75.0–98.0		28/32 (87.5)	71.0–96.5		27/32 (84.4)	67.2–94.7	
Rectum	16/19 (84.2)	60.4–96.6		16/19 (84.2)	60.4–96.6		14/19 (73.7)	48.8–90.9	
Pathological type			1.000			1.000			.336
Conventional adenocarcinoma	41/46 (89.1)	76.4–96.4		40/46 (87.0)	73.7–95.1		38/46 (82.6)	68.6–92.2	
Mucinous adenocarcinoma	4/5 (80.0)	28.4–99.5		4/5 (80.0)	28.4–99.5		3/5 (60.0)	14.7–94.7	
Differentiation grade			.037			.046			.002
Well/moderately differentiated	39/42 (92.9)	80.5–98.5		38/42 (90.5)	77.4–97.3		37/42 (88.1)	74.4–96.0	
Poorly differentiated	6/9 (66.7)	29.9–92.5		6/9 (66.7)	29.9–92.5		4/9 (44.4)	13.7–78.8	

Data are presented as the number of positive cases/total cases and sensitivity (%), with exact 95% confidence intervals. Confidence intervals were calculated using the Clopper–Pearson method. P values represent comparisons between the 2 categories within each clinicopathological characteristic using the chi-square test or Fisher’s exact test, as appropriate. “Both positive” was defined as simultaneous positivity for SATB2 and CDX2.

CDX2 = caudal type homeobox 2, CI = confidence interval, SATB2 = special AT-rich sequence-binding protein 2.

Marker sensitivity did not differ significantly according to age. Among patients aged ≤ 65 years (n = 26), the sensitivities of SATB2, CDX2, and the “both positive” rule were 88.5% (23/26; 95% CI, 69.8–97.6%), 84.6% (22/26; 95% CI, 65.1–95.6%), and 80.8% (21/26; 95% CI, 60.6–93.4%), respectively. Corresponding sensitivities among patients aged > 65 years (n = 25) were 88.0% (22/25; 95% CI, 68.8–97.5%), 88.0% (22/25; 95% CI, 68.8–97.5%), and 80.0% (20/25; 95% CI, 59.3–93.2%). None of the age-related comparisons was statistically significant (all *P* > .05).

Similarly, no significant differences were observed according to sex or primary tumor site. Among male patients (n = 29), the sensitivities of SATB2, CDX2, and the “both positive” rule were 86.2%, 82.8%, and 75.9%, respectively, compared with 90.9%, 90.9%, and 86.4% among female patients (n = 22; all *P* > .05). In colon adenocarcinomas (n = 32), the corresponding sensitivities were 90.6%, 87.5%, and 84.4%, whereas in rectal adenocarcinomas (n = 19), they were 84.2%, 84.2%, and 73.7%, respectively; these differences were also not statistically significant.

With respect to pathological type, conventional adenocarcinomas (n = 46) showed sensitivities of 89.1% for SATB2, 87.0% for CDX2, and 82.6% for the “both positive” rule. In mucinous adenocarcinomas (n = 5), the corresponding sensitivities were 80.0%, 80.0%, and 60.0%. No statistically significant differences were identified between the 2 pathological types. However, the estimates for mucinous adenocarcinoma had wide 95% CI because of the small number of cases.

Tumor differentiation was the only clinicopathological characteristic significantly associated with marker sensitivity. Among well- or moderately differentiated tumors (n = 42), SATB2 sensitivity was 92.9% (39/42; 95% CI, 80.5–98.5%), compared with 66.7% (6/9; 95% CI, 29.9–92.5%) among poorly differentiated tumors (*P* = .037). CDX2 sensitivity similarly decreased from 90.5% (38/42; 95% CI, 77.4–97.3%) to 66.7% (6/9; 95% CI, 29.9–92.5%; *P* = .046). The largest reduction was observed for the “both positive” rule, for which sensitivity decreased from 88.1% (37/42; 95% CI, 74.4–96.0%) in well- or moderately differentiated tumors to 44.4% (4/9; 95% CI, 13.7–78.8%) in poorly differentiated tumors (*P* = 0.002; Table 7).

## 4. Discussion

In this study, we evaluated the value of SATB2 and CDX2 in the diagnosis of colorectal adenocarcinoma (CRC) and analyzed the diagnostic efficacy of these 2 markers when used individually and in combination. The results of the study showed that the expression of SATB2 and CDX2 in colorectal adenocarcinoma was significantly higher than that in the control group, with positivity rates of 88.2% and 86.3% in the colorectal adenocarcinoma group, respectively. These findings are consistent with previous studies, indicating that these 2 markers possess high sensitivity and specificity and are effective tools for the diagnosis of colorectal cancer.^[13,14]^ Notably, SATB2 used alone demonstrated the best diagnostic efficacy, with an AUC value of 0.916 and a specificity of 93.0%, highlighting its superiority in the diagnosis of colorectal adenocarcinoma.^[8,15]^

When SATB2 and CDX2 were used in combination, the results were further optimized. Under the “either positive” strategy, the sensitivity reached 94.1% with a negative predictive value of 94.7%, making it highly suitable for the exclusion diagnosis of colorectal cancer. In contrast, the “both positive” strategy showed the most outstanding diagnostic accuracy, with a specificity of 98.6%, a positive predictive value of 97.6%, and the strongest comprehensive diagnostic ability (AUC = 0.938), making it suitable for confirmation diagnosis.^[9,16]^ These results indicate that the combined strategies can enhance sensitivity while maintaining high specificity, making them applicable to more complex diagnostic scenarios in clinical practice.

It is worth noting that CDX2 had the lowest specificity (60%) when differentiating gastric adenocarcinoma, which may be related to the relatively high false-positive rate of CDX2 in gastric adenocarcinoma.^[17]^ However, when the “both positive” strategy was employed, this strategy demonstrated extremely high diagnostic reliability across all differential scenarios, suggesting that the combined use of SATB2 and CDX2 can effectively compensate for the limitations of a single marker and improve overall diagnostic accuracy.^[18]^

The study also found that in poorly differentiated colorectal adenocarcinoma, the sensitivity of both SATB2 and CDX2 decreased significantly, suggesting that SATB2 and CDX2 may not provide sufficient sensitivity in the diagnosis of poorly differentiated carcinomas.^[19]^ This finding provides an important tip for clinical practice, indicating that when dealing with poorly differentiated colorectal cancer, it may be necessary to combine other markers or diagnostic methods to improve accuracy.

The limitations of this study lie in its retrospective design, which may introduce selection bias, and the relatively small sample size, particularly in the poorly differentiated carcinoma subgroup where the number of specimens was limited. This may affect the generalizability of the results. Future research could consider conducting prospective cohort studies, increasing the sample size of patients with poorly differentiated carcinomas, and further validating the practical effectiveness of the combined application of SATB2 and CDX2 in clinical diagnosis.

Because this study used a retrospective case-control design with predefined numbers of colorectal and non-colorectal adenocarcinoma cases, PPV and NPV estimates should be interpreted cautiously. These values reflect diagnostic performance within the present study cohort and may differ in clinical practice where the prevalence of colorectal adenocarcinoma among suspected cases varies. Therefore, sensitivity, specificity, and receiver operating characteristic-based measures may provide more generalizable information regarding marker performance.

In conclusion, SATB2 and CDX2, as immunohistochemical markers, possess significant clinical application value in the differential diagnosis of colorectal adenocarcinoma. SATB2 demonstrates high specificity when used alone, while the combined use of both markers can provide higher sensitivity and accuracy. Although the sensitivity of both decreases when facing poorly differentiated carcinomas, combined detection remains an effective strategy to improve diagnostic accuracy. Therefore, the combined application of SATB2 and CDX2 can serve as an important tool in the clinical diagnosis of colorectal cancer, especially in complex or difficult differential diagnosis scenarios.

## Author contributions

**Conceptualization:** Liping Wang, Hongjun Li, Liping Feng.

**Data curation:** Liping Wang, Hongjun Li, Liping Feng.

**Formal analysis:** Liping Wang, Hongjun Li, Liping Feng.

**Funding acquisition:** Liping Feng.

**Investigation:** Liping Feng.

**Writing – original draft:** Liping Feng.

**Writing – review & editing:** Liping Feng.
